# Obituary for Jan Valentyn van der Merwe (19 December 1943 – 25 September 2015)

**Published:** 2016-03-28

**Authors:** W Ombelet

Professor Jan Valentyn Van der Merwe passed away on Friday, 25 September, at the age of 71.

**Figure:**
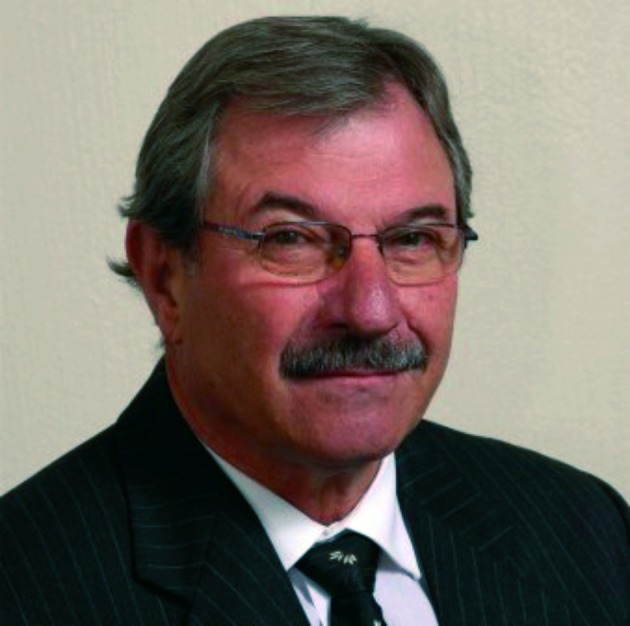


Prof Jan’s illustrious career began after he completed a research fellowship at Duke University in the USA, and returned to South Africa as Head of the Department of Obstetrics and Gynaecology at Pretoria University. In this role, Prof Jan pioneered the establishment of an assisted reproduction unit in South Africa, which was ultimately responsible for the first successful IVF procedure in South Africa.

Many Flemish gynaecologists who worked in Pretoria as a trainee for one or more years will remember Prof Jan as a charismatic and inspirational leader, the one you never forget, the one you admired. I am still very grateful he convinced me to join his infertility- team in 1984.

In the late 80s he became the Dean of the Faculty of Medicine at the University of Pretoria, Prof Jan played a leading role within the Coordinating Committee of Deans of Medical Faculties of South Africa, of which he was the Chairman from 1989 to 1994. His involvement and leadership were particularly significant during the period of change within the healthcare industry during SA’s transition to democracy, and subsequently. Prof Jan was appointed by the then-Minister of Health, Dr N Zuma, as a member of the Implementation Support Group for Academic Hospitals of the National Department of Health.

Prof Jan was also involved in a number of statutory councils such as the National Population and Medicine Control Councils, the Council for Academic Hospitals and the Health Professions Council of SA. He played a vital role in the development of numerous policies related to both the private and public health sector. As the founder of the Board of Health Executives (BHE) in the late 1990s, Prof Jan spearheaded the development of the BHE Scientific Medicine Formulary, a first-of-its-kind document. The scientific formulary represented a major advancement in how medicine was prescribed.

His involvement at Universal Healthcare resulted in several innovations, the most significant being the impact of basing healthcare funding decisions on scientific evidence and international best practice, with a caring approach.

Prof Jan was honoured with a Lifetime Achievement Award for his considerable contribution to medicine at the Healthcare Funders’ inaugural Titanium Awards in August 2015, soon before he died.


Willem Ombelet


